# Differential Diagnosis of Abdominal Tuberculosis in the Adult—Literature Review

**DOI:** 10.3390/diagnostics11122362

**Published:** 2021-12-15

**Authors:** Sinziana Ionescu, Alin Codrut Nicolescu, Octavia Luciana Madge, Marian Marincas, Madalina Radu, Laurentiu Simion

**Affiliations:** 1 1st Clinic of General Surgery and Surgical Oncology, Bucharest Oncology Institute, 022328 Bucharest, Romania; sinzianaionescu30@gmail.com (S.I.); dr.simion.laurentiu@gmail.com (L.S.); 2 Department of Surgery, “Carol Davila” University of Medicine and Pharmacy, 050474 Bucharest, Romania; 3 Roma Medical Center for Diagnosis and Treatment, 011774 Bucharest, Romania; 4 Pathology Department, Bucharest Oncology Institute, 022328 Bucharest, Romania; madalinar405@gmail.com

**Keywords:** tuberculosis, differential, diagnosis, peritonitis, lymphadenopathy, granuloma, nodules, cyst, laparotomy, laparoscopy

## Abstract

Tuberculosis (TB) is a public health issue that affects mostly, but not exclusively, developing countries. Abdominal TB is difficult to detect at first, with the incidence ranging from 10% to 30% of individuals with lung TB. Symptoms are non-specific, examinations can be misleading, and biomarkers commonly linked with other diseases can also make appropriate diagnosis difficult. As a background for this literature review, the method used was to look into the main characteristics and features of abdominal tuberculosis that could help with differentiation on the PubMed, Science Direct, and Academic Oxford Journals databases. The results were grouped into three categories: A. general features (the five forms of abdominal tuberculosis: wet and dry peritonitis, lymphadenopathy, lesions at the level of the cavitary organs, lesions at the level of the solid organs), B. different intra-abdominal organs and patterns of involvement (oesophageal, gastro-duodenal, jejunal, ileal, colorectal, hepatosplenic, and pancreatic TB with calcified lymphadenopathy, also with description of extraperitoneal forms), and C. special challenges of the differential diagnosis in abdominal TB (such as diagnostic overlap, the disease in transplant candidates and transplant recipients, and zoonotic TB). The study concluded that, particularly in endemic countries, any disease manifesting with peritonitis, lymphadenopathy, or lesions at the level of the intestines or solid organs should have workups and protocols applied that can confirm/dismiss the suspicion of abdominal tuberculosis.

## 1. Introduction

In the beginning of the 21st century, tuberculosis was declared by the WHO to be ‘a global emergency’. Many aspects reinforce and underline this statement: (1) the fact that tuberculosis continues to be an important communicable disease and a leading cause of morbidity and mortality; (2) tuberculosis kills (also) young adults, thereby impacting on the workforce; (3) open cases of tuberculosis infect the patient’s environment; and (4) TB is known to be a social burden on the family, society, and nations.

Tuberculosis is a common systemic infection with the bacteria *Mycobacterium tuberculosis*, which is primarily found in the lungs and causes caseous inflammation in lung tissue and other organs. Tuberculosis is an infectious disease that spreads via the air. Each person with active tuberculosis can infect 10 to 15 persons each year if they are not cured. Over two billion people are infected with the tuberculosis bacteria. In his or her lifetime, one out of every ten of those people will contract active tuberculosis. HIV-positive people are at a substantially higher risk. Tuberculosis is a poverty-related illness, with the vast majority of people killed in poor countries, and with Asia accounting for more than half of all deaths.

Multidrug-resistant tuberculosis (MDR-TB) is a kind of tuberculosis that does not respond to first-line drug therapies. It is exceedingly tough to treat, a fact proven in more than 50 countries.

More than 100 facultative saprophytes and entities that are acid-fast mycobacteria, but that do not cause tuberculosis or leprosy, have been discovered in research. These mycobacteria, also known as atypical mycobacteria (ATM), can cause a variety of illnesses, including septic arthritis, abscesses, and skin and bone infections. They can also harm the lungs, gastrointestinal tract, lymphatic system, and other organs.

Tuberculosis is an endemic disease in developing countries, as mentioned, and, due to the wide spread of acquired immunodeficiency syndrome (AIDS), it might represent a problem in developed countries, as well. Only around one-fifth of patients diagnosed with abdominal TB have pulmonary disease. The causative organisms involved are *Mycobacterium tuberculosis hominis*, *Mycobacterium bovis*, and Atypical mycobacterium, such as *Mycobacterium avium intracellulare*, which are usually identified in patients suffering from AIDS. The transmission can occur in various ways: via ingestion of infected milk or cough droplets, via hematogenous spread from a distant source, via lymphatic spread, or via direct extension from adjacent organs. Abdominal TB can affect the peritoneum and its reflections, the gastrointestinal tract, the lymphatic system, or the solid organs. In the present literature review, the most common forms are described, in correlation with the main organs and also in different clinical situations, with an emphasis on different means to successfully perform a differential diagnosis.

## 2. Materials and Methods

**Figure 1:**
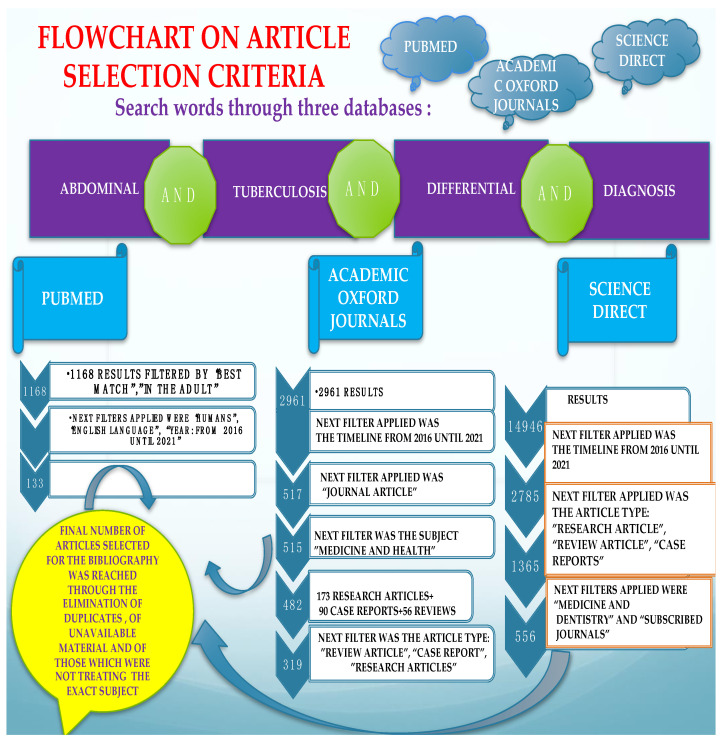
Flowchart illustrating the selection criteria employed in the current review.

**Figure 2:**
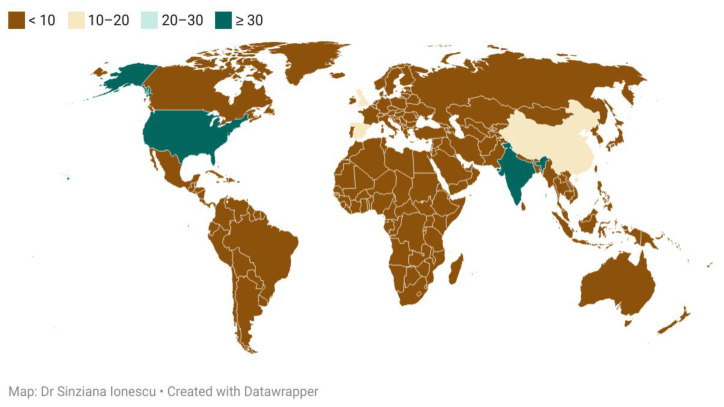
Choropleth map with consulted articles and their country of provenience.

Various searches performed on the PubMed, Oxford Journals, and Science Direct databases, as well as the supplementary information found, were used to compile this literature review. The keywords employed were: “Abdominal AND tuberculosis AND differential AND diagnosis”, with consequent findings including abstracts and full-text papers of research articles, reviews and systematic reviews, and case reports. Additional filters were “in the human species” and “written in English”, with a focus on the most recent findings (2016–2021), relevant and quoted papers indexed on the subject, referring to the adult, without taking into account pediatric cases, or the special physiologic state of pregnancy. Papers published before 2016 were only quoted if the research performed afterwards on the particular subject was scarce. Figure 1. depicts the flowchart of selection criteria for the articles used in the present review. After the detailed study, the results were underlined by the authors, prioritizing the most relevant information, regarding clinical, paraclinical, and epidemiological particularities of abdominal TB and its differential diagnosis in the adult patient. The retrieved information is either presented in the text or in the tables, which show similar studies in each subsection. A world choropleth map (Figure 2) is depicted in showing the number of articles consulted, grouped in relation to the main author’s country of provenience (Table 1).

## 3. Results

In this results section, the research is grouped into three categories: (A) general features, regarding the diagnosis of abdominal tuberculosis; (B) different intra-abdominal organs and their involvement; and (C) special challenges of the differentiation of abdominal TB.

### 3.1. General Features Regarding the Diagnosis of Abdominal Tuberculosis

The research conducted found several main ideas regarding the manifestations and the differential diagnosis workup. The clinical forms of abdominal TB can be grouped, with the help of the algorithm described by [1], into five categories: first, peritonitis with its two forms: (a) “wet”, requiring proper diagnostic aspiration, and laparoscopy, and (b) “dry/fixed”, which requires endoscopy. Second, there is lymphadenopathy, which can be further described with ultrasound-guided aspiration and laparoscopy. Third, there is the intestinal set of lesions that are seen with endoscopy, and fourth, there are the lesions described at the level of the solid organs, where a biopsy can be performed. As a last resort, in cases that have not concluded any diagnosis, regardless of the set of investigations performed, there is the indication for laparotomy, with consequent pathology examination from the tissue fragments retrieved.

**Figure 3:**
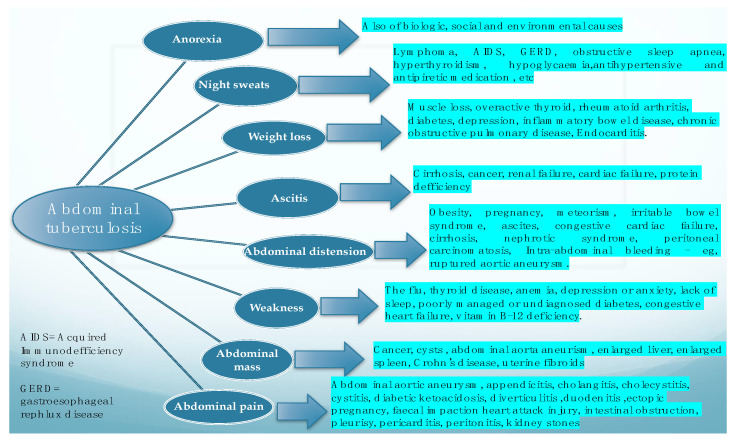
Main clinical manifestations of abdominal tuberculosis, and the main differential for each of them.

In a literature review performed by [2] in 2004, in which the authors looked at the main features of the cases suffering from abdominal tuberculosis, the most frequently found features were ascites, weight loss, weakness, abdominal mass, abdominal pain, abdominal distention, anorexia, and night sweats. This research concluded that abdominal TB should be considered in all cases with ascitis, and that the authors’ experience suggested that PCR test of the ascitic fluid obtained by ultrasound-guided fine-needle aspiration is a reliable method for diagnosis and should at least be attempted before surgical intervention. Figure 3. depicts the main clinical symptoms of abdominal tuberculosis and also basic data for their differentiation.

Another study, which revisited an older review performed by [3], looked into the diagnostic challenges of abdominal tuberculosis, as it does not present with specific features and is, thus, regarded as a great mimicker. Imaging is described as highly important in the early recognition of the disease.

In “stubborn” cases, presenting with difficult differential diagnostic issues, with many investigations and little orientation towards an incriminating pathogen, a review done by [4] indicated the importance of laparoscopy in the diagnosis of abdominal tuberculosis. Furthermore, apart from mentioning common literature themes on the subject, such as the fact that abdominal pain and weight loss are commonly encountered and that TB presents non-specifically, this literature search emphasized the fact that the most consistent lab findings (in a percentage of more than 90%) were low hemoglobin and raised C-reactive protein. In the general context that the Mantoux tuberculin test was positive only in a small percentage, and where Ziehl Neelsen staining of the ascitic fluid was also negative in all patients, laparoscopy proved useful in yielding a diagnosis in 92% of the cases.

An analysis made in 1994 [4] reviewed the features of abdominal TB and found that 10 to 30% of the patients with lung TB also had abdominal involvement, and that the incidence was higher in immigrants from developing countries and patients with AIDS. The idea was underlined further by the fact that in HIV-infected patients the disease is of a rapidly progressing nature and often of a fatal outcome, as diagnosis is difficult and delayed, while treatment is mostly medical but can also be surgical.

**Figure 4:**
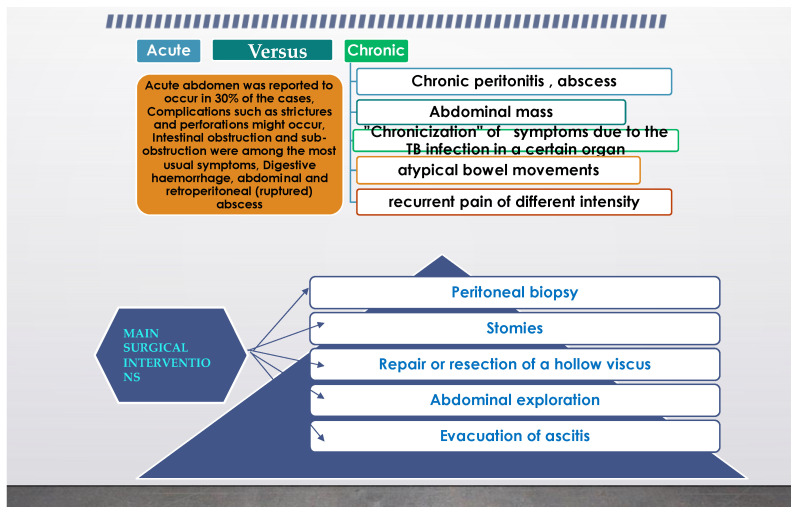
Common surgical setting characteristics in abdominal tuberculosis.

From the previous paragraph, it can be inferred that abdominal TB can also represent a surgical problem, and in another review from [5] it was stated that even an astute surgeon can find a challenge in the diagnosis. It is not uncommon for extrapulmonary TB to present as an acute abdomen in surgical emergencies, such as perforations and obstructions of the bowel. Moreover, abdominal TB, in its different forms was found more often as an etiology for a chronic abdomen. The paper concluded that abdominal TB should always be considered one of the differential diagnoses of acute or chronic abdomen in endemic areas. TB peritonitis can be caused by reactivation of a latent TB focus, by the discharge of caseous material from diseased lymph nodes, or TB salpingitis in females. In the “wet” ascitic type, which is found in the majority of cases, there is a large amount of fluid that can be free or loculated. In the “fibrotic” fixed type, there is a mesenteric and omental thickening, and masses can be found with matted bowel loops and sometimes loculated ascitis. In the “dry” or plastic-type, there are caseous nodules, a fibrous peritoneal reaction, and dense adhesions. The most commonly encountered surgical settings in a patient with abdominal tuberculosis are summarized in Figure 4.

When TB associates with ascitis, ultrasound can detect even a small amount and show fine, multiple strands of fibrin, septations, and debris. A computed tomography exam can show the high density of fibrin and cellular debris or water density in the initial transitive phase, or even the fat fluid level due to chylous ascitis. A magnetic resonance imaging exam can show delayed enhancement (15–20 min) after i.v. contrast.

When studying the multifaceted lesions of peritoneal carcinomatosis and its mimickers in a review from 2014 [6], the main aspect underlined was that invasive peritoneal disease included more than just peritoneal carcinomatosis, and a rigorous CT analysis may also show pseudomyxoma peritonei, peritoneal lymphomatosis, tuberculosis, peritoneal mesothelioma, diffuse peritoneal leyomyomatosis, and benign splenosis.

Radiologic imaging should also be included in the workup for abdominal tuberculosis; CT is preferred, because it allows for the assessment of lymphadenopathy, ascites, and peritoneal and solid organ involvement.

Additional imaging modalities may be beneficial: ultrasound may be used to detect lymphadenopathy, ascites, and peritoneal and intestinal thickening, while a barium enema may be used to detect mucosal ulcerations, strictures, and ileocecal valve incompetence.

An abdominal X-ray can be helpful in determining air–fluid levels (during the presentation of intestinal obstruction) and liver and/or ureter calcifications.

### 3.2. Different Intra-Abdominal Organs and Their Involvement

The differential diagnosis between abdominal TB and other important diseases was taken into consideration in various studies, considering a particular organ or form in which TB manifests, as described in the following subsections.

#### 3.2.1. Oesophageal TB

Oesophageal TB is described as secondary to advanced forms of pulmonary or mediastinal tuberculosis. The sources can be tuberculous laryngitis, caseating lymph nodes, vertebral body infection, and also the lymphatic/hematogenous route. Barium study shows extrinsic compression by enlarged nodes, ulcerations, smooth strictures, mucosal irregularity, traction diverticula, and fistulae. CT assists in identifying the peripheral extension of the disease.

According to the research carried out by [7], oesophageal TB can manifest as dysphagia. Various other details related to oesophageal TB, from the scarce literature on the subject, can be found in Table 2.

#### 3.2.2. Gastroduodenal TB

Gastroduodenal tuberculosis presents in a variety of ways, including stomach abdominal pain and dyspepsia, as well as obstruction of the gastric outlet. Upper digestive endoscopy can produce ulcer lesions, narrowing, and fistulas, which might require further investigations and treatment procedures, as was presented by [11]. Table 3 further describes the comparative features of gastroduodenal tuberculosis.

CT imaging of the stomach can reveal the hypertrophic features of tuberculous pyloric stenosis in its late stages. Although the presence of a sinus tract and fistula is uncommon, they are indicative of tuberculosis. The gastric antrum and distal body are the most frequently involved structures. The hypertrophic form can exhibit severe and diffuse wall thickening on imaging. Antral narrowing can occur as a result of ulceration and fibrosis.

The duodenum is most frequently affected by the extrinsic compression caused by adjacent lymphadenopathy, which manifests as obstruction and is easily detected on CT scans. Additionally, the duodenum may exhibit intrinsic hypertrophic involvement, which is visible on CT as thickening of the duodenal walls. Ulcers can result in strictures and fistulae, similar to the case of the stomach, which are readily visible in barium studies.

#### 3.2.3. Jejunal, Ileal, and Colonic TB

The small bowel mesentery is commonly involved in patients with peritoneal TB. Investigations have shown nodular lesions, solid or cystic, forming from lymph nodes or abscess, and mesenteric thickening (of more than 15 mm for diagnosis purposes), while the mesentery appears hypoechoic, due to fat deposition from lymphatics. An ultrasound exam can find a stellate sign with fixed loops of bowel and with the mesentery standing out as spikes radiating from the root of the mesentery. The “club sandwich” or “slice bread sign” describes the focal ascitis between radially oriented bowel loops, due to exudation from inflamed bowel loops or ruptured lymph nodes. Computed tomography shows a thickened mesentery, as has been previously mentioned, with increased vascularity and tethering of bowel loops, forming an abdominal mass.

Intestinal TB is mainly located in the ileocecal area, in isolation or as part of a multifocal involvement. Findings of a barium study can be labelled, first of all, as “highly suggestive”, when one or more of the following features are seen: deformed ileocecal valve with dilated terminal ileum, contracted cecum with an abnormal ileocecal valve and/or terminal ileum, stricture of the ascending colon with shortening and involvement of the ileocolic region. Other traits are “suggestive”, but less specific, such as a contracted cecum, ulcerations or narrowing of the terminal ileum, stricture of the ascending colon, multiple areas of dilation, and narrowing or matting of small bowel loops. A CT scan of the ileocecal region can contribute further information on the aspect of this bowel segment and its appearance, the main differential is with Crohn’s disease, as can be seen in the overlap subsection of this article. Some studies affirmed that with TB, night sweats are more common, while with Crohn’s, diarrhea and perianal disease are to be found more often, as described by [14].

In the situation of an immunosuppressed patient, enteritis can also be caused by *Mycobacterium avium intracellulare* complex infection (MAC), which can occur in cases of MAC disseminated infection, in patients with AIDS, or with transplant recipients. Diagnosis is established by isolating the organisms from blood cultures, and when MAC enteritis is suspected stool cultures are useful. MAC infection can mimic Whipple disease, as was found by [15].

The large bowel is involved in around 9-10% of cases without small bowel involvement. A scan can show segmental long/short involvement, spiculation, spasm, rigidity and ulceration, inflammatory polyps, perforation, fistulae, and pericolic abscess.

#### 3.2.4. Rectal TB

Rectal tuberculosis is a rare occurrence and data with more info can be found in Table 4. 

Due to its unique epidemiological characteristics, it requires specialized medical care, and surgery is rarely needed.

Diagnosis is frequently delayed.

Rectal tuberculosis patients are known to have some risk factors, including concomitant diseases linked with a variety of defective host–defense mechanisms, such as acquired immunodeficiency syndrome or complement insufficiency.

Rectal TB is more prevalent in females than males.

Hematochezia is the most frequently presenting symptom encountered.

A definitive diagnosis requires histopathologic evidence of Mycobacterium tuberculosis bacillus.

Rectal tuberculosis is treatable with antituberculous medication if a correct diagnosis has been obtained.

Surgery is indicated in cases of diagnostic impasses, intractable disease, and complications.

The review of the literature referred to was undertaken by [16].

#### 3.2.5. Liver and Splenic TB

Liver and spleen involvement is common in milliary TB or in the portal vein in gastrointestinal lesions.

The form encountered can be micronodular, as is the case of milliary TB, with the ultrasound showing a bright liver and spleen and the computed tomography showing small nodules, which are not easily seen. If found, hepatosplenomegaly can be homogenous or heterogeneous.

The macronodular form is another variation of this type and is seen on ultrasound as hypoechoic lesions, sometimes hyperechoic, with or without calcification, and, un-commonly, a “honeycomb” aspect is found. Computed tomography shows a low-density lesion (15–45 Hounsfield units), with a minimal central enhancement and moderate peripheral enhancement. Magnetic resonance imaging shows a hypointense lesion on T1-weighted images with a hypointense rim and a iso- to hyperintense lesion on T2-weighted images with a less intense rim.

Various studies, among which we refer to [21], have described the use of elastography in abdominal tuberculosis, as a means of diagnosis of portal hypertension of a benign etiology.

During a review looking at the splenic forms of TB, [22] found that splenic involvement occurs more in males, in an age group of 19–53, that fever of unknown origin is the typical presentation, and that it can present with small splenomegaly, splenic abscess, associated with ascitis or not.

Studies on key-findings in the literature searched on the topic of “splenic tuberculosis” were mentioned in Table 5. 

#### 3.2.6. Pancreatic TB. Approach to Cystic Lesions. Pancreatic Cysts

Pancreatic tuberculosis is another uncommon entity that mimics a pancreatic tumor.

Half of the instances identified in the reviewed literature were associated with pulmonary tuberculosis.

Frequently presenting with multi-cystic pancreatic mass (81%), the most prevalent anatomic regions of the pancreas were the head (73%), tail (18%), and body (9%) of the pancreas.

According to a study conducted by [27], clinical suspicion and an appropriate diagnostic method, including FNAB of the pancreatic lesion, are required to prevent undergoing a needless laparotomy.

X-ray imaging of pancreatic tuberculosis reveals a hypodense lesion with an uneven edge, and pancreatic enlargement with/without peripancreatic node expansion.

According to [28], cystic lesions discovered in and surrounding the peritoneal cavity can frequently be difficult to diagnose, due to an extensive overlap.

When approaching a cystic lesion, it is critical to analyze the following imaging characteristics: cyst content, locularity, wall thickness, and the existence of internal septa, solid components, calcifications, or any related enhancements.

While conclusive diagnosis is not always feasible following imaging, a careful examination of the imaging appearance, location, and connection to neighboring tissues can assist in narrowing the differential diagnosis. Table 6 summarizes the referred studies and their main research features on pancreatic tuberculosis.

#### 3.2.7. Calcified Lymphadenopathy and Tuberculous Lymphadenitis

One of the most frequent indications for endoscopic ultrasound-guided tissue biopsy is to elucidate the source of suspicious lymphadenopathy. Even if most cases are benign and auto-limiting, patients with deep-seated lymph nodes living in TB-endemic areas, or with suspected malignancy, require tissue diagnosis to guide treatment. TB lymphadenitis can vary from increased numbers of normal size lymph nodes, to massive con-glomerate/matted nodes, to peri adenitis. The sites of lymph nodes involving secondary to lymphatic drainage from the bowel are mesenteric periportal, anterior pararenal, upper para-aortic, and lesser omental regions. Hematogenous spread might lead to the following sites of infected lymph nodes: mesenteric, lesser omental, anterior pararenal, and upper and lower paraaortic. Direct spread can lead to infection of lymph nodes from adjacent organs. According to the findings from a study by [32], tuberculous lymphadenitis can appear on a CT scan as: (a) in the first stage with homogenous enhancement, (b) in the second stage as central caseous necrosis and peripheral rim enhancement, and (c) in the final stage as fibrosis and calcifications.

Magnetic resonance imaging has proven useful in showing the relationship of the lymph nodes to the vessels, ducts, and, also for differentiating necrotic peripancreatic nodes from an adenocarcinoma of the head of the pancreas, as will be seen further on.

Several types of research of tuberculous endemic regions reported between approximately one-third and one half of cases as tuberculous adenitis, with a diagnostic yield of 89%. In patients presenting with a high clinical suspicion of TB, the study reported tuberculous adenitis in over 70% of abdominal lymphadenopathies. Fine needle aspiration guided by endoscopic ultrasound (EUS-FNA) [33] had a sensitivity of 86%, a specificity of 100%, and 91% negative predictive values for TB. The first rationale of the study was that TB remains a serious menace to public health, but hepatic and pancreatic TB are rare, and, therefore, the preoperative diagnosis of pancreatic TB remains a great challenge. The lessons learned in the research conducted by [34] were connected to calcification, in both pancreatic lesions and peripancreatic lymph nodes, as a suggestion of pancreatic TB, rather than pancreatic malignancy.

Tuberculous lymphadenitis can also be found in the clinical setting of a severe complication such as is the case of obstructive jaundice secondary to TB, an extremely rare entity that can be caused by TB enlargement of the head of the pancreas, TB lymphadenitis, TB stricture of the biliary tree, or a TB mass in the retroperitoneum, as will be seen in another subsection. The investigations employed were ultrasound, computed tomography, ERCP and CEA, and CA 19-9, which were normal, as well as PCR testing [34].

#### 3.2.8. Primary Gallbladder Tuberculosis

As the gallbladder is rarely involved in abdominal TB as a primary organ, extensive research literature on the subject has been limited to case reports, which present it as a rare differential among the more common gallbladder pathologies, and it is the pathology exam that establishes the diagnosis [35].

#### 3.2.9. Pelvic TB, Retroperitoneal and Extraperitoneal Space Lesions

Urinary tract TB may be found in consequence of the hematogenous spread of infection from the primary source elsewhere, most commonly past or present pulmonary infection, followed by bone or joint TB. Initially, both the kidneys may be involved and organisms are lodged in the glomerular and peritubular capillary network. Most of the lesions appear initially heal without sequelae, and only a few progress to clinical and radiological anomalies. The symptoms vary between frequency, burning, urgency, hematuria, renal pain, weight loss, fatigue, and fever. Intravenous pyelography (IVP) can be employed and is best at detecting early findings (70% of cases show unilateral disease progression and radiological abnormality, and IVP only picks up the lesion when it ulcerates at the level of the calyx), while ultrasound and computed tomography help with the evaluation of late changes. Standard X-ray can offer details of calcifications, and if the hydronephrotic kidney becomes non-functioning, then extensive dystrophic putty-like calcification may form a cast of the kidney, which is known as auto-nephrectomy. Other transformations that can hint at TB are phantom calyx, amputated calyx, hiked-up pelvis, gross hydronephrosis, extensive parenchymal destruction, beaded ureter, ureteric stricture, tubercular cystitis with edema, and thimble bladder. A CT-scan is indicated in patients with normal or equivocal findings on IVP and ultrasound, and it can depict multiple small poorly-enhanced nodules, uneven calix-ectasis and calcification pattern, and thickening of the ureter.

The theoretical context depicts female genital TB as a form of extrapulmonary TB in developing countries, where it is an important cause of infertility, ectopic pregnancy, and miscarriage. Therefore, in the above-mentioned setting, elevated Ca-125 with abdominal pelvic mass must raise suspicion for TB [36].

Regarding male genital tract TB, delay in diagnosis may lead to complications, such as infertility and perineal scrotal sinuses. Imaging plays an important part in depicting the findings and helping with the differential diagnosis. MRI is optimal in the evaluation of the prostate, seminal vesicles, and ejaculatory ducts, while high-resolution ultrasonography is the best modality for assessing the epididymis, testis scrotum, and vas deferens.

Psoas abscesses generally arise from a contiguous intra-abdominal or pelvic infectious process or hematogenous spread of bacteria. The serum beta-human chorionic gonadotropin has been used to detect normal or ectopic pregnancy, and it can also be used in following up carcinomas. In a case presented by [37] the biopsy of the left psoas demonstrated metastatic or infiltrating poorly-differentiated carcinoma with the secretion of Beta human chorionic gonadotropin. The subsequent pathological examination of the neoplasm showed the same pathologic morphology. The important issue raised by the authors was that clinicians need to sharpen their awareness of the potential malignant carcinomas mimicking a psoas abscess.

TB was also described as an infectious cause of primary hypoadrenalism [38].

### 3.3. Special Challenges in the Differentiation of Abdominal TB

#### 3.3.1. Diagnostic Overlap

In a review article [39] on the diagnosis and management of Crohn’s disease in populations at high risk for tuberculosis, it was noted that differentiating Crohn’s disease from intestinal tuberculosis in endemic areas is difficult, due to the overlapping clinical, radiological, endoscopic, and histological characteristics of both conditions.

Additionally, high rates of latent tuberculosis increase the likelihood of reactivation, once treatment for established Crohn’s disease is initiated.

The researchers concluded that a diagnosis of Crohn’s disease in persons at risk for tuberculosis should be made, only after a thorough interpretation of clinical symptoms, abdominal imaging, and systematic endoscopic and histological testing.

Newer approaches for diagnosing latent tuberculosis in this context remain to be verified, and guidelines for treating latent tuberculosis in this situation require clarifying.

#### 3.3.2. TB in Transplant Candidates and Transplant Recipients

According to a study conducted by [40], latent tuberculosis infection (LTBI) is defined as the presence of immunological sensitization to Mycobacterium tuberculosis (MTB) in the absence of clinical or radiological indications of active disease.

Lymph nodes (thoracic, cervical, mesenteric, and retroperitoneal) and from the lung (apices and bases) were collected from 628 persons, ranging in age from infancy to old age, and MTB was isolated from animals inoculated with 96 individuals’ tissues (15.3%).

Additional evidence supporting the presence of MTB in the lung, liver, and kidney of latently infected individuals comes from natural experiments, in which tissues from donors with LTBI were transplanted into immunocompromised recipients, with numerous studies indicating that MTB has a widespread anatomical distribution in latently infected individuals.

#### 3.3.3. Zoonotic Tuberculosis zTB

According to a study conducted by [41], zoonoses account for 60% of all new infectious diseases, demonstrating the group’s importance. A significant zoonosis is tuberculosis (TB) caused by *Mycobacterium bovis*, also known as zoonotic TB (zTB). Historically, zTB has been associated with extrapulmonary tuberculosis, transmitted mostly through the intake of unpasteurized milk, and the route of infection affects the location of the initial lesion.

In general, active disease is caused by the reactivation of a latent infection, as a result of immunosuppression, the latter associated with stress or old age, and occurring even in treated patients who are deemed to have recovered.

Certain factors, including chemotherapy, diabetes, nephrosis, malnutrition, host vulnerability, and socioeconomic status, can all affect the progression and form of zTB, as described by [42].

Immunosuppression induced by HIV infection increases the risk of latent tuberculosis progressing to active illness, in addition to representing an adverse prognostic factor [43].

#### 3.3.4. Challenges Imposed by HIV–TB Coinfection

While antiretroviral medication (ART) is critical in the fight against tuberculosis (TB) and HIV coinfection, the TB-associated immune reconstitution inflammatory syndrome complicates the treatment (TB-IRIS). Immune-mediated deterioration of TB pathology can take the form of paradoxical TB-IRIS, unmasking TB-IRIS, or CNS (central nervous system) TB-IRIS, depending on the TB disease site and treatment state at the time of the beginning of ART. Each type of TB-IRIS has its own diagnostic and therapy implications. Along with the well-known roles of CD4 T cells and macrophages, recent investigations have highlighted the involvement of neutrophils and T cell subtypes in TB-IRIS pathogenesis. While corticosteroids remain the only trial-supported medication for the prevention and management of TB-IRIS, biologic medicines that directly target the immunological pathology are gaining traction. TB-IRIS, particularly in its unmasking form, is still poorly understood, and further research is needed to verify biomarkers for diagnosis, as found by [44].

#### 3.3.5. Pathogenicity and Complexity of the Immune Response in Case of TB Infection

Only 10% of the persons infected with TB are free of the pathogen, which can escape during infection and remain latent in existing lesions, making the infection difficult to manage. In response to perturbations in the immune response, a loss in immunity can lead to MTB resuscitation and active tuberculosis. MTB infection triggers a complex immunological response, and the germs have sophisticated immune escape strategies, for instance the inhibition of (a) phagolysosome maturation and acidification, (b) of the oxidative stress and the function of reactive oxygen, and (c) of the reactive nitrogen intermediates. As a result, it is critical to understand these pathways, in order to diagnose and treat the disease effectively, according to a study presented by [45].

Regardless of the suspicion levels indicated by various investigations, the microbiological diagnosis of tuberculosis is a necessary component of confirmation, and its key characteristics are listed in Table 7.

Moreover, inspired by the pioneering fact that Robert Koch used animal model systems to test his hypotheses, while identifying the *Bacillus anthracis* and *Mycobacterium tuberculosis,* model systems are still employed by researchers, even today. Their advantages can reveal details regarding disease etiology and introduce therapy regimes of critical importance in the understanding of these conditions. The different in vitro and in vivo model systems used to research the above-mentioned two illnesses are, according to [54] grouped into several categories: (a) employed in the study of the disease mechanism, such as is the case of zebra fish, guinea pig, mouse and rat, and cell lines; (b) employed in drug/vaccine testing, which compels all the systems (the ones mentioned before plus non-human primates and rabbit); (c) employed in the study of virulence, as happens with cell lines, mouse and rat, zebra fish and guinea pig; furthermore, other parameters studied can be (d) immunological, in which non-human primates, mouse and rat, rabbit and guinea pig systems are employed.

## 4. Discussion

Tuberculosis can affect virtually any organ and system in the human body and has a wide variety of radiologic appearances, which can mimic numerous other diseases and malignancies. Abdominal tuberculosis is a common extrapulmonary manifestation, with the incidence increasing in the context of incomplete treatment, multidrug-resistant strains, and HIV prevalence. Patient morbidity and mortality can be significantly diminished with early diagnosis, and, especially in endemic countries, abdominal tuberculosis should be a priority, and the differential diagnosis and the multifaceted features of the disease should be always in the clinician’s mind, to intervene and start treatment, even before the complications emerge, as is dictated by the patient’s best interests.

## Figures and Tables

**Table 1:** List of countries, depicting the number of articles found during the search and grouped according to the first author’s country of provenience.

Country Name	Number of Articles	Country Name	Number of Articles
Belgium	1	United States of America	36
Cameroon	1	India	32
Colombia	1	China	17
Democratic Republic of Congo	1	United Kingdom	14
Ecuador	1	Spain	11
Indonesia	1	Netherlands	9
Ireland	1	D. P. R. of Korea	8
Kosovo	1	Japan	7
New Zealand	1	France	6
Nigeria	1	Germany	6
Peru	1	Pakistan	5
Portugal	1	Brazil	4
Qatar	1	Australia	3
R. B. de Venezuela	1	South Africa	3
Romania	1	Turkey	3
Rwanda	1	United Arab Emirates	3
Saudi Arabia	1	Canada	2
Sudan	1	Denmark	2
Tanzania	1	Israel	2
Thailand	1	Italy	2
Uganda	1	Mexico	2
Zambia	1	Singapore	2

**Table 2:** Oesophageal tuberculosis and a comparison between the findings from different studies.

Name of the Study, Journal Where It Was Published, and Year	Investigations Used, Purpose of the Study	Findings	Number of Cases
Kocaman, Turk. J. Gastroenterol., 2013 [8]	Endosonography and elastography in the diagnosis of esophageal tuberculosis	Esophageal tuberculosis	Case report
V. D. Plat, Journal Of Surgery, 2017 [9]	Comparing endoscopic repair with surgical repair in brochoesophageal fistulas	Fistulas were caused by postoperative complications or pulmonary TB	16 cases
S.K. Jain, The American Journal of Gastroenterology, 2002 [7]	Upper endoscopy Pathology exam Cytology	Pts with esophageal TB constituted 0.5% of pts with dysphagia and 1.3% of all pts with abnormal esophagoscopic findings	12 cases
M.C. Borges, Dig. Dis. Sci., 2009 [10]	Evaluate the role of PCR in the etiology of ulcers in HIV-1 infected pts	96 biopsies from HIV infected pts were processed by specific PCR	79 cases
S.Jain, Acta Cytol., 1999 [11]	To study the utility of endoscopic cytology in the diagnosis of esophageal TB in clinically unsuspected cases	228 cases of esophageal lesions	8 cases

**Table 3:** Gastroduodenal tuberculosis: examples of literature studies and their key features.

Review	Key-Features
P. Chaudhary, Indian Journal of Tuberculosis, 2019 [12]	Gastric TB is rare It mimics gastric carcinoma Responds well to conservative management Requires surgery for complications
M. Barat, Diagnostic and Interventional Imaging, 2017 [13]	Duodenal Tb is rare, the disease more commonly affects the ileocecum, colon, and jejunum, while more than 90% of duodenal TB was also found to have co-infections with other parts of the intestine On CT, duodenal Tb presents as strictures, extrinsic compression, polypoidal intraluminal mass, and duodenal ulcerations, associated with multiple hypoattenuating lymph nodes
S. Srisajjakul, Clinical Imaging, 2016 [14]	Duodenitis Aortoduodenal fistula

**Table 4:** Rectal tuberculosis: literature studies and their key features.

Study	Key-Features
Poras Chaudhary, Indian Journal of Tuberculosis, 2021 [17]	Rare, commonly misdiagnosed, curable with chemotherapy, surgery for complications
Jan Rakinic, Advances in Surgery, Volume 52, Issue 1, 2018 [18]	Anorectal fistulas
Puri, A.S., Dis. Colon Rectum, 1996, [19]	Rectal Tb can cause strictures
Chaudhary, A., Dis. Colon Rectum, 1986 [20]	Two cases of rectal strictures
Patil, S., Indian J. Tuberc., 2013 [21]	Rectal prolapse

**Table 5:** Splenic tuberculosis: literature studies and their key features.

Study	Form of TB Encountered in the Study
MDCT Findings of splenic pathology, Sangster, 2021 [23]	Microabscess, splenomegaly
A review of the cysts of the spleen, Khan, 2016 [24]	Peliosis
Improving diagnosis of atraumatic splenic lesions, Ricci, 2016 [25]	Calcified granuloma and peliosis
Splenic tuberculosis: a comprehensive review of literature, Gupta, 2018 [26]	Small splenomegaly, abscess+/- ascitis
Hypersplenism secondary to splenic tuberculosis, Ais, 1993 [27]	Hypersplenism

**Table 6:** Pancreatic TB.

Main Author	Main features of the research	Main aspects followed
Ray et al., JOP, 2012 [29]	Pancreatic and peripancreatic nodal tuberculosis in immunocompetent patients: report of 3 cases	Description of pancreatic TB with contrast-enhanced ultrasound
Irfan M. Monaldi Arch. Chest Dis. 2013 [30]	Tb pancreatitis complicating with a ruptured splenic artery pseudoaneurysm	Emergency laparotomy for haemorrhage
D. Cruz S. JOP, 2003 [31]	Pancreatic TB	FNA biopsy

**Table 7:** Diagnostic methods in tuberculosis, as per WHO consolidated guidelines on tuberculosis, Module 3: Diagnosis [46].

Method, Category		Main Features
A. Microscopy		First line is auramine phenol staining, relying on the autofluorescence of the bacterial wall, followed in some labs by Ziehl-Neelsen staining [47,48]
B. Culture		Gold standard for diagnosis, a positive culture is achieved in 2/3 of cases of extrapulmonary TB (automated liquid culture or solid culture Lowenstein Jensen) [49,50]
C. Immunological methods		Tuberculin skin testing or interferon gamma release assay [51,52]
D. DNA-based methods		Nucleic acid amplification tests or whole genome sequencing tests, used for the detection of mycobacteria, identification of the most common mutations, strain typing
D.1. Initial tests for diagnosis of TB with drug-resistance detection	Xpert MTB/RIF assay	The Xpert MTB/RIF assay is a cartridge-based automated test that uses real-time polymerase chain reaction (PCR) on the GeneXpert platform to identify MTBC and mutations associated with RIF resistance directly from sputum specimens in less than 2 h.
	2. Xpert MTB/RIF Ultra assay	The Xpert MTB/RIF Ultra assay (hereafter called Xpert Ultra) uses the same GeneXpert platform as the Xpert MTB/RIF test and was developed to improve the sensitivity and reliability of detection of MTBC and RIF resistance
	3. Truenat MTB, MTB Plus and MTB-RIF Dx assays	The Truenat MTB and MTB Plus assays use chip-based real-time micro PCR for the semiquantitative detection of MTBC directly from sputum specimens and can report results in under an hour. The assays use automated, battery- operated devices that extract, amplify, and detect specific genomic DNA loci.
	4. Moderate complexity automated NAATs	The moderate complexity of the automated NAATs class of tests includes rapid and accurate tests for the detection of pulmonary TB from respiratory samples.
D.2. Initial tests for diagnosis of TB without drug-resistance detection	TB-LAMP assay	The TB-LAMP assay is designed to detect MTBC directly from sputum specimens. This is a manual assay that provides results in less than 1 hour, does not require sophisticated instrumentation, and can be used at the peripheral health centre level, given biosafety requirements similar to those for sputum-smear microscopy. TB-LAMP does not detect resistance to anti-TB drugs.
	2. Urine LF-LAM	The urine LF-LAM is an immunocapture assay based on the detection of the mycobacterial LAM antigen in urine; it is a potential point-of-care test for certain populations being evaluated for TB. Although the assay lacks sensitivity, it can be used as a fast, bedside, rule-in test for HIV-positive individuals, especially in urgent cases, where a rapid TB diagnosis is critical for the patient’s survival.
D.3. Follow-on diagnostic tests for detection of additional drug resistance	Low complexity automated NAATs for detection of resistance to INH and second-line anti-TB drugs	The “first in class” product for low complexity automated NAATs for detection of resistance to INH and second-line anti-TB drugs is the Xpert MTB/XDR Assay (Cepheid, Sunnyvale, USA). This test uses a cartridge designed for the GeneXpert instrument to detect resistance to INH, FQs, ETO, and second-line injectable drugs (AMK, kanamycin and capreomycin)
	2. LPA s	LPAs are a family of DNA strip-based tests that detect mutations associated with drug resistance. They do this either directly, through binding DNA amplification products (amplicons) to probes targeting the most commonly occurring mutations (MUT probes), or indirectly, inferred by the lack of binding of the amplicons to the corresponding wild-type probes.
	3. High complexity reverse hybridization NAAT	The “first in class” product for this class is the GenoScholar PZA-TB (Nipro, Osaka, Japan) for the detection of resistance to PZA. The GenoScholar PZA-TB test is based on the same principle as the FL-LPA and SL-LPA but requires the use of a large number of hybridization probes to cover the full *pncA* gene (>700 base pairs [bp])
E. New methods, such as biosensors		The greatest potential and widespread use of biosensors for diagnosing Mycobacterium tuberculosis and drug resistance belong to DNA electrochemical biosensors (isoniazid and rifampin strains).Biosensors are increasingly being used to detect resistant strains of anti-TB antibiotics with high sensitivity and accuracy, and the speed of these sensory methods is also critical [53].

## Data Availability

Not applicable.

## Abbreviations

TB	Tuberculosis
MTB	*Mycobacterium tuberculosis*
HIV	Human Immunodeficiency Virus
MDR-TB	multi-drug resistant tuberculosis
ATM	Atypical mycobacteria
AIDS	Acquired Immune Deficiency Syndrome
i.v.	Intravenous
Pts	Patients
CT	computed tomography
MRI	Magnetic Resonance Imaging
FNAB	fine needle aspiration biopsy
FNA	fine needle aspiration
CA 19-9	carbohydrate antigen Ca19-9
CEA	carcinoembryonic antigen
ERCP	endoscopic retrograde cholangiopancreatography
PCR	polymerase chain reaction
IVP	intravenous pyelography
TB-IRIS	TB associated immune reconstitution inflammatory syndrome that complicates treatment
ART	antiretroviral medication
